# Reflective Optics Design for an LED High Beam Headlamp of Motorbikes

**DOI:** 10.1155/2015/503171

**Published:** 2015-04-15

**Authors:** Peng Ge, Xiang Wang, Yang Li, Hong Wang

**Affiliations:** ^1^Engineering Research Center for Optoelectronics of Guangdong Province, School of Physics and Optoelectronics, South China University of Technology, Guangzhou 510640, China; ^2^Department of Mechatronics Engineering, School of Mechanical & Automotive Engineering, South China University of Technology, Guangzhou 510640, China

## Abstract

We propose a reflective optics design for an LED motorbike high beam lamp. We set the measuring screen as an elliptical zone and divide it into many small lattices and divide the spatial angle of the LED source into many parts and make relationships between them. According to the conservation law of energy and the Snell's law, the reflector is generated by freeform optics design method. Then the optical system is simulated by Monte Carlo method using ASAP software. Light pattern of simulation could meet the standard. The high beam headlamp is finally fabricated and assembled into a physical object. Experiment results can fully comply with United Nations Economic Commission for Europe (ECE) vehicle regulations R113 revision 2 (Class C).

## 1. Introduction

With the development of LED technology, its optical efficiency becomes higher and higher. It is reported that recently Cree Company announced that their white LED's experimental optical efficiency reached up to 300 lm/W. And with the development of materials, its thermal conductivity will not be a serious problem anymore. LED's advantages in life span, high optical efficiency, energy savings, good reliability, fast reaction, small size, good thermal resistance, and cost reduction, will promote LED's wide application in every industry.

Some literatures reported researches on LED motorcar headlamps [1–13]. However most of them presented just simulation results. And to our best known, we have not found literatures about LED's application in motorbike's headlamps. In this paper, we propose an LED high beam headlamp design based on a reflective optics. We use mesh generation method to divide the beam pattern on the measuring screen according to the United Nations Economic Commission for Europe vehicle regulations (ECE) R113 revision 2 (Class C) [14]. And then we divide the spatial angle of the LED source according to the conservation of energy principle. Finally we use Snell's law to obtain the freeform surface of the reflector. The rays radiating from the LED source will be redistributed by the freeform reflector. By adjusting the coefficient parameters, the light pattern of the high beam headlamp will reach the ECE standard R113 revision 2.

## 2. Methodology

**Figure 1:**
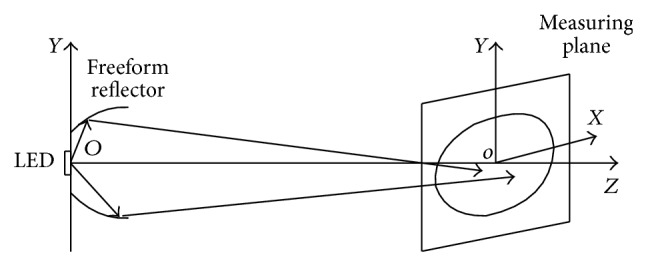
Sketch of the projection relationship.

According to the ECE standard R113 revision 2 (Class C) the light distribution of the high beam headlamp should have enough illumination at the test points [14]. The center point of the illumination plane should be located in the maximum illumination zones. A long ellipse beam pattern will be formed by the optics of the high beam headlamp, as shown in Figure 1. LED source is located in the center of the reflector. The emitting surface of the LED is paralleled with the measuring plane. Most of the rays radiating from the LED source are reflected by the inner surface and toward the measuring screen. Another portion of the rays is emitting directly to the measuring plane.

**Figure 2:**
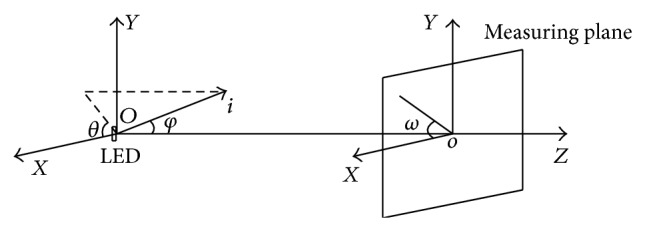
Coordinates setup.

The distance between the LED source and the measuring screen is set as *d* = 25 meters. Total luminous flux is *Q*. The center intensity is *I*_0_ = *Q*/*π*. We select the center of the LED chip as the original point of Cartesian coordinates as shown in Figure 2. The emitting surface of the LED chip is set as *XOY* plane. *z*-axis is vertical to *XOY* plane and passes through the original point. Plane *XOZ* is horizontal. The plane paralleling to *XOY* plane and crossing the *z*-axis at point *o* is called measuring plane. The distance between point *O* and point *o* is 25 meters. Point *o* is the center of the measuring screen. The angle *φ* is the angle between the emitting rays and the positive *z*-axis. *θ* is the angle between *x*-axis and projection of emitting rays in the *XOY* plane. *ω* is the angle between the *x*-axis and the line that links any points in the measuring plane and point *o*.

**Figure 3:**
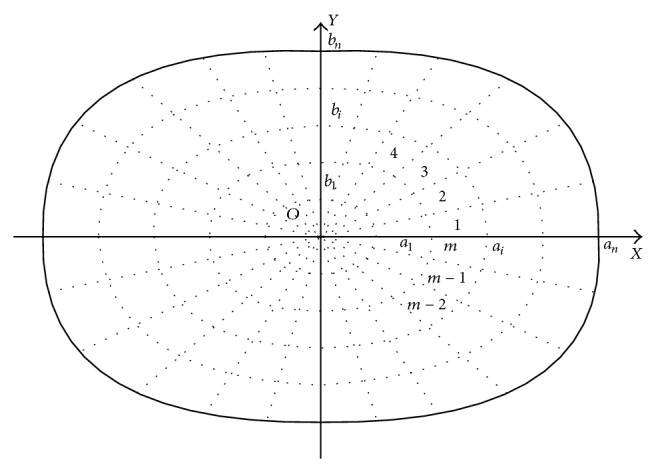
Division of the measuring zones.

Then we set the measuring zones as an ellipse. The semimajor axis is *a*, and the semiminor axis is *b*. We discrete the coordinates of the measuring screen. The semimajor axis and the semiminor axis are divided into *n* parts. *a*_*i*_ and *b*_*i*_ represent the *i*th part of the division, in which 0 < *i* ≤ *n*. We draw the ellipse with the center of the illumination plane as the ellipse center, *a*_*i*_, *b*_*i*_ as the semimajor axis and the semiminor axis. In this way, the illumination plane is divided into *n* elliptical ribbons. Then the *ω* (0 ≤ *ω* ≤ 360°) is divided into *m* segments, and *ω*_*j*_ represents the *j*th part of the division of *ω*. In the measuring screen, the rays that originate from the *o* and form an angle of *ω*_*j*_ with *x*-axis divide every elliptical ribbon into *m* segments, as shown in Figure 3. So the illumination plane is divided into *m* × *n* small lattices.

**Figure 4:**
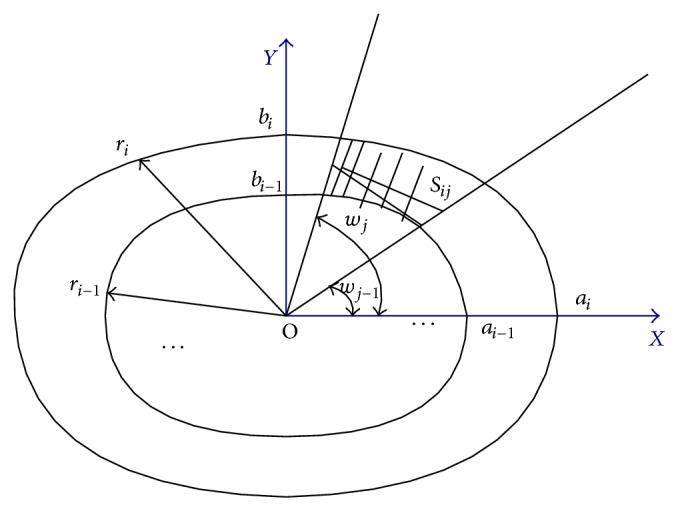
The sketch of the area of the ribbon.

Considering ellipse equation in polar coordinates as shown in Figure 4, the ellipse can be expressed as

(1) $$\begin{array}{c} \;\frac{r^{2}\cos^{2}\omega }{a^{2}}+\frac{r^{2}\text{si}{\text{n}}^{2}\omega }{b^{2}}=1, \end{array}$$

in which *r* is the distance between the ellipse and the origin point. So *r* can be expressed as

(2) $$\begin{array}{c} r^{2}=\frac{a^{2}b^{2}}{\cos^{2}\omega \cdot b+\text{si}{\text{n}}^{2}\omega \cdot a}. \end{array}$$

The area in polar coordinates can be expressed as

(3) $$\begin{array}{c} S=\overset{\omega_{j}}{\underset{\omega_{j-1}}{\int }}\frac{1}{2}r^{2}d\omega . \end{array}$$

So the area of some ribbon in Figure 4 is expressed as

(4) Sij=∫wj−1wj12ri2dω−∫wj−1wj12ri−12dω=12∫wj−1wjai2bi2cos⁡2ω·bi+sin2ω·ai     −ai−12bi−12cos⁡2ω·bi−1+sin2ω·ai−1dω.

Energy of each lattice is expressed as

(5) EQ=E·kij·∫ωj−1ωj12·ai2·bi2cos⁡2ω·bi2+sin2ω·ai2     −ai−12·bi−12cos⁡2ω·bi−12+sin2ω·ai−12dω,

in which *E* · *k*_*ij*_ represents the illumination value. We set *E* a predefined value according to the ECE regulation R113 revision 2. *k*_*ij*_ is used to control the value of specific zones in the illumination plane to form the predetermined light distribution, in which 0 ≤ *k*_*ij*_ ≤ 1. The value of *k*_*ij*_ should be decided upon the requirement of the illumination intensity. For the brightest areas, *k*_*ij*_ should be chosen between 0.9 and 1, and for the darkest areas, *k*_*ij*_ should be chosen between 0 and 0.1.

Correspondingly, we discrete the spatial angular of the part of the LED source which participates in the high beam distribution. We divide *φ* into *n* parts. *φ*_*i*_ represents the *i*th part and corresponds with *a*_*i*_ and *b*_*i*_. Similarly, we divide *θ* into *m* parts. *θ*_*j*_ represents the *j*th part and corresponds with *θ*_*j*_ and *ω*_*j*_. Before reflection, luminous flux of each angular is

(6) $$\begin{array}{c} E_{q}=\overset{\theta_{j}}{\underset{\theta_{j-1}}{\int }}\overset{\phi_{i}}{\underset{\phi_{i-1}}{\int }}I_{0}\cdot \cos\phi \cdot \sin\phi \;d\phi \;d\theta . \end{array}$$

Because the distance between the LED source and the target plane is too far, that is 25 meters, the illumination caused by directly emitting from the source is actually very small. So we omit this portion. The energy conservation principle is expressed as

(7) $$\begin{array}{c} E_{Q}=E_{q}. \end{array}$$

Combine with the above equation (1) to (3), we can solve *φ*_*i*_ and *θ*_*j*_.

Then we can solve the normal vectors of the points on the above curve surfaces. Tangent lines can be calculated according to the normal vectors. Finally coordinates on the curves can be solved by calculating the tangent lines intersecting with the incident lines. The vector form of refraction law is represented as

(8) $$\begin{array}{c} {\left[1+n^{2}-2\cdot n\cdot \left(\vec{{\text{Out}}_{}}\cdot \vec{\mathrm{In}}\right)\right]}^{1/2}\cdot \vec{N_{}}=\;\vec{{\text{Out}}_{}}-n\cdot \vec{\mathrm{In}} \end{array}$$

in which $\vec{\mathrm{In}}$ is unit vector of incident lines,  $\vec{{\text{Out}}_{}}$ is unit vector of outgoing rays, $\vec{N_{}}$ is normal vector. Index of refraction in the reflective system is *n* = 1.

**Figure 5:**
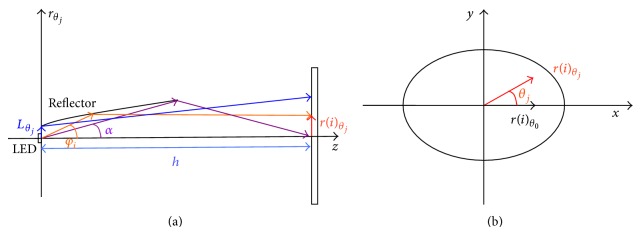
The sketch of the reflector.

In Figure 5(a), *α* is the angle between the *z*-axis and the line which crosses the center of the LED source and the edge of the reflector. We consider rays with angle *θ*_*j*_ as an example. Because the reflector is symmetrical with *z*-axis, we only consider *θ*_*j*_ ∈ [0, *π*/2]. *φ*_*i*_ represents the angle between the *z*-axis and the *i*th ray hitting on the reflector, in which *φ*_*i*_ ∈ [*α*, *π*/2]. *L*_*θ*_*j*__ means the distance between the center of the LED source and the point on the reflector when *z* coordinate is equal to zero. *r*(*i*)_*θ*_*j*__ in Figure 5(b) means the distance between the center of the target plane and the edge point corresponding to the rays with angle *φ*_*i*_ and *θ*_*j*_. We obtained

(9) $$\begin{array}{c} r{\left(i\right)}_{\theta_{0}}=a_{i},\\ r{\left(i\right)}_{\theta_{j}}=a_{i}b_{i}\sqrt{b_{i}^{2}\cos^{2}\theta_{j}+a_{i}^{2}\text{si}{\text{n}}^{2}\theta_{j}}. \end{array}$$

**Figure 6:**
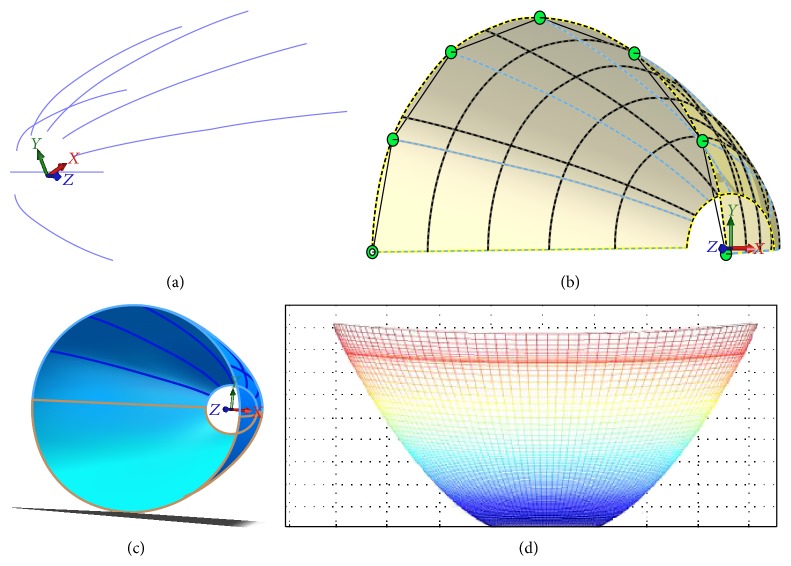
The calculation of the reflector.

**Figure 7:**
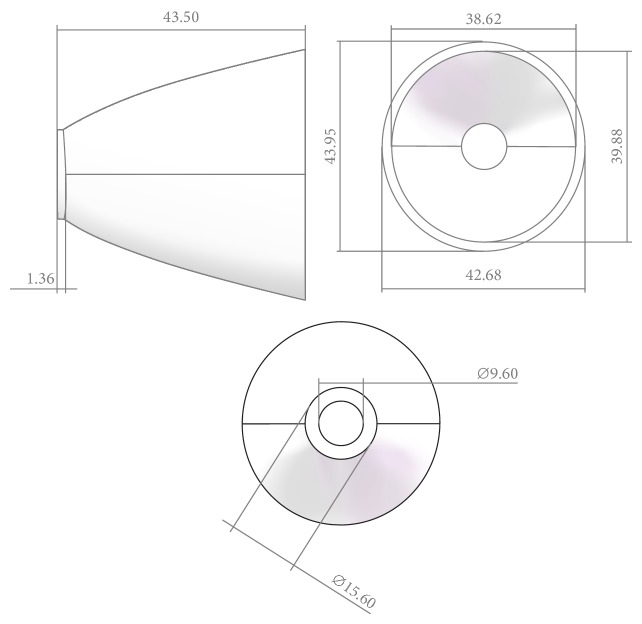
Entity and size of the high beam's reflector.

In the calculation process, we should initialize the parameters, such as the aperture of the bottom margin of the trapezium. We set semimajor axis *a* = 6, semiminor axis *b* = 2, *h* = 25, *L*_*θ*_*j*__ = 0.0048, *φ*_0_ = *π*/2, and *α* = 0.4189. And we set *ϕ*_*i*_ = *α* as the terminal condition of the iteration process. The margin curves of the reflector can be fixed according to the initial points, as shown in Figure 6(a). Then the whole freeform surface can be calculated according to the points on the margin curves. The surface of the reflector can be generated by lofting the discrete points introduced into the mechanical modeling software, as shown in Figure 6(b). By mirror imaging, we get the whole reflector as shown in Figure 6(c).  Figure 6(d) shows the whole freeform curves on the reflector. Finally the entity and the size of the reflector are as shown in Figure 7. The height of the reflector is 43.5 mm, and the aperture of the reflector is 38.62 mm.

## 3. Simulation

**Figure 8:**
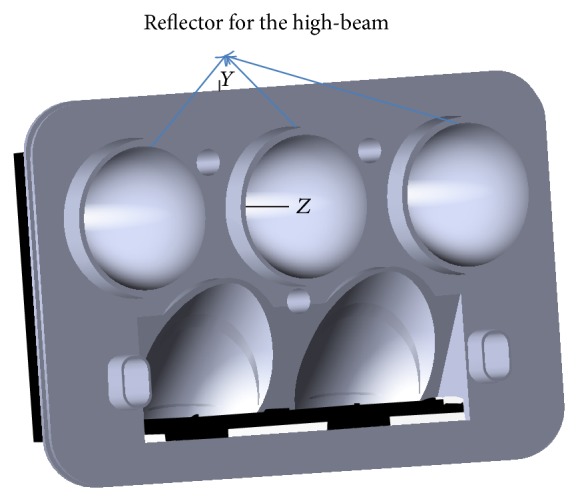
Optical setup in the simulation.

**Figure 9:**
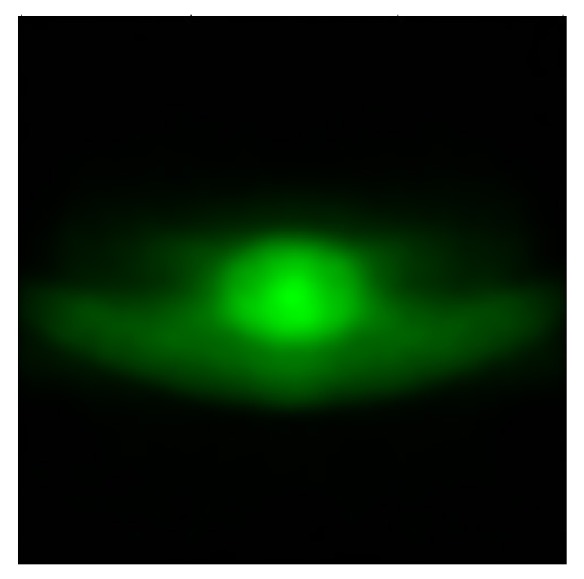
The light distribution of the simulation result.

The reflector entity is introduced into optical simulation software, which traces the rays by using Monte Carlo method. We choose LUW Q9WP made by Osram Corporation as the LED source and set the total flux of single LED chip as 300 lm. In order to obtain the required total illumination, we use three same LED chips as the source and three same reflectors as the optics. So the total flux in simulation is 900 lm. The illumination distribution is measured on the plane 25 meters in front of the LED source. Total illumination received in the measuring screen is 792.9 lm. So the optical efficiency is about 88%. We adjust *k*_*ij*_ in order to obtain the ideal light spots. Optical setup for the simulation is shown in Figure 8. Simulation result is shown in Figure 9. The light patterns satisfy the ECE regulation R113 revision 2 (Class C).

## 4. Experiments

**Figure 10:**
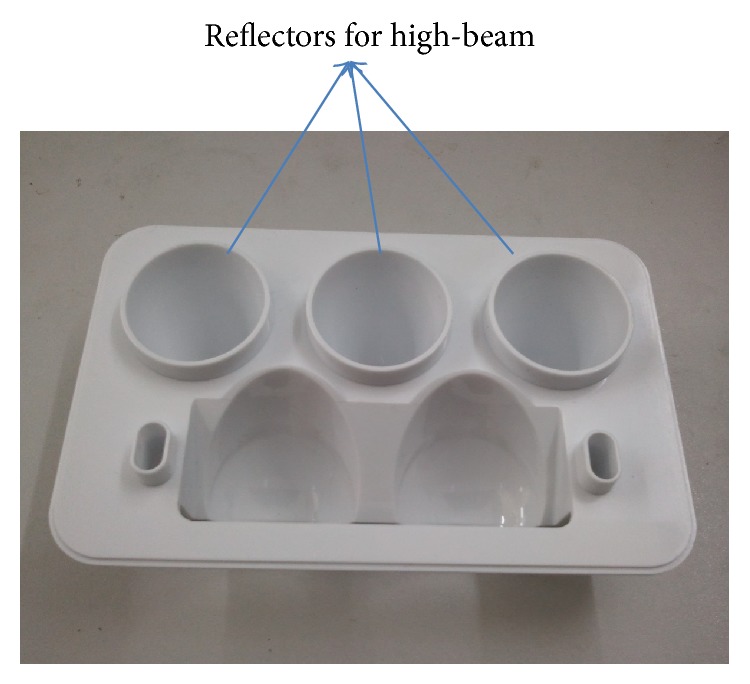
Mold of the designed headlamp.

**Figure 11:**
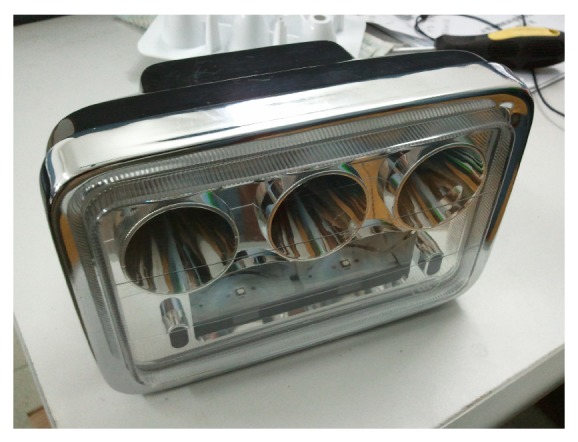
Assembled motorbike's headlamp.

**Figure 12:**
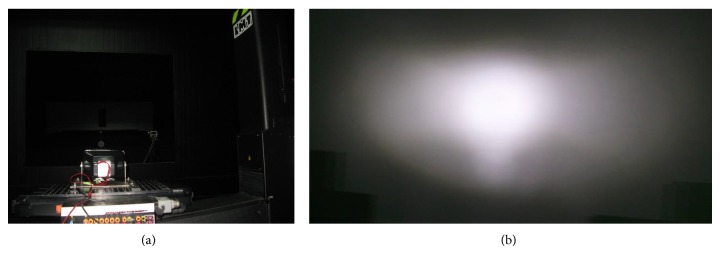
(a) Measured configuration and (b) light pattern of the test results.

Finally the designed entity was processed and fabricated into a mold for experimental test, as shown in Figure 10. By electroplating and assembling, we assembled motorbike's optical system as shown in Figure 11. The total power of the high beam is 9 W, including three LED chip models. Then it was measured by LMT-400 system in an authoritative test organization, located in Guangzhou City. Test configuration is shown in Figure 12(a). The light distribution is shown in Figure 12(b). The measured results are shown in Table 1. All test points have passed the ECE regulation R113 revision 2 (Class C).

## 5. Conclusion

In conclusion, we design an LED high beam headlamp for motorbikes based on a free-form reflector. Simulation and experimental results show that our headlamp can both reach the ECE regulation R113 revision 2 (Class C). The optical system is easy to be fabricated. It can be widely used in the motorbikes' headlamp for LED sources.

## Figures and Tables

**Table 1:** The measured results.

Number	Function	Min	Max	Value	H	V	Unit	N.O.K
1	HV	>0.8*E*max	180	37.238	0.00°	0.00°	lx	
2	H-3R	12.8	—	18.06	3.00°	0.00°	lx	
3	H-3L	12.8	—	22.291	−3.00°	0.00°	lx	
4	H-6R	4.16	—	8.172	6.00°	0.00°	lx	
5	H-6L	4.16	—	7.975	−6.00°	0.00°	lx	
6	H-9R	2.56	—	5.038	9.00°	0.00°	lx	
7	H-9L	2.56	—	4.766	−9.00°	0.00°	lx	
8	H-12R	0.8	—	4.315	12.00°	0.00°	lx	
9	H-12L	0.8	—	4.285	−12.00°	0.00°	lx	
10	2U-V	1.28	—	12.952	0.00°	0.00°	lx	
11	4D-V	0.3*E*max	0	17.039	0.00°	−4.00°	lx	
12	Maximum	32	180	42.062	−0.30°	−1.30°	lx	
13	Color point	—	—	34.457	0.00°	0.00°	lx	
